# The unsung heroes of traditional healers in healing and traditional medicine in dementia care for Indigenous populations in North America: an exploration of a compassionate, community-led care model

**DOI:** 10.3389/fpubh.2026.1800362

**Published:** 2026-04-23

**Authors:** Hom Lal Shrestha, Lucy Shrestha, Rosellen M. Rosich, Michael Splaine, Art Petahtegoose

**Affiliations:** 1 Faculty of Health, York University, Toronto, ON, Canada; 2 School of Kinesiology and Health Sciences, Laurentian University, Sudbury, ON, Canada; 3 Department of Psychology, College of Arts and Sciences, University of Alaska Anchorage, Anchorage, AK, United States; 4 Splaine Consulting, Washington, DC, United States; 5 McEwen School of Architecture, Laurentian University, Sudbury, ON, Canada

**Keywords:** community-led, culturally-safe dementia care, health equity, holistic healing, Indigenous, traditional healers, two-eyed seeing framework

## Abstract

This paper explores the critical role, experience, and wisdom of traditional healers in providing culturally-safe dementia care for Indigenous populations in North America, particularly as the prevalence of dementia in these communities continues to rise. It highlights the varying perceptions of dementia across cultures; for instance, while Western medicine often views it as a disease, many Indigenous cultures interpret it as a spiritual transition within the life cycle. A literature review conducted from 2000 to 2025 indicates that the marginalization of traditional healers within formal healthcare systems significantly hinders the delivery of culturally sensitive dementia care. Community-led models illustrate that the integration of traditional healing methods with contemporary practices is vital for offering respectful care to Indigenous people living with dementia. These models are grounded in concepts such as co-design, relational accountability, and “Two-Eyed Seeing,” which establish traditional healers as crucial partners in the care process. The World Health Organization (WHO) formally recognized traditional healers as “community stakeholders” for dementia care and prevention globally. However, without the engagement and empowerment of traditional healers in the screening and diagnosis process of dementia, the implementation of the WHO Global Action Plan (2017–2025) on the public health response to dementia will be hindered, as these traditional healers are essential community stakeholders and allied partners for dementia care and prevention worldwide. The paper advocates for systemic reforms to support traditional healers and promote Indigenous self-determination in dementia care and calls for the development of medical education specifically tailored for traditional healers to establish a biocultural dementia care model that encompasses community support systems, spiritual approaches, a dementia-friendly environment, language preferences, and Indigenous culture, including arts, sciences, and philosophy.

## 1 Introduction

Indigenous Peoples have engaged in traditional medicine and healing practices for tens of thousands of years across many nations globally. These practices are grounded in holistic worldviews that perceive health as a balance among the physical, mental, emotional, spiritual, relational, and land-based aspects of life (1). The definitions of traditional healing and traditional medicine are interchangeable. According to Struthers et al. (2), Dr. Alvord, the first Navajo female surgeon, defines a traditional healer in Navajo culture medicine as someone who sees a person not simply as a body, but as a whole being. Mind and spirit are seen as connected to other people, families, communities, and even the planet and the universe. Indigenous men and women traditional healers, also known as medicine men/women, practice the art of traditional healing within their communities and may also provide services across tribes and to non-native people (2, 3). On the other hand, a study by Hart (2007) reveals that Indigenous knowledge and the ways of connecting to all of creation are fundamentally relational. This knowledge is holistic and personal (subjective), inherently social (dependent on interrelations), and profoundly influenced by local ecosystems. It is also intergenerational, encompassing both spiritual and physical dimensions, and relies heavily on Elders for its development and transmission (4).

Additionally, traditional healing has also been defined as “practices designed to promote mental, physical, and spiritual well-being that are based on beliefs that go back to the time before the spread of Western ‘scientific’ biomedicine” by the Royal Commission on Aboriginal Peoples (1996). When Aboriginal Peoples in Canada talk about traditional healing, they include a wide range of activities, from physical cures using herbal medicines and other remedies to the promotion of psychological and spiritual well-being using ceremony, counseling, and the accumulated wisdom of Elders (5) (p. 325).

However, colonial histories, epistemological injustices, and institutionalized hierarchies of knowledge have long marginalized traditional healing within Western biomedical systems, despite its enduring presence. However, Redverse (6) stresses and concludes that traditional medicine and medical science can coexist with mutual respect and understanding for the benefit of both Indigenous and non-Indigenous patients worldwide (6). Furthermore, the care and support of individuals experiencing dementia is an increasingly significant global health priority, presenting profound cultural and ethical challenges. In North America, this challenge is particularly acute for Indigenous communities, because the dominant biomedical model of dementia often stands in stark contrast to Indigenous worldviews regarding health, aging, community and the mind.

At present, Indigenous communities are facing a dementia crisis caused by significant inequity. In Canada, the number of Indigenous peoples living with dementia is projected to increase by 273% by 2050, compared to an expected rise of 187% in the non-Indigenous population (7). Similarly, 34% of the Indigenous population is affected by dementia, with projections indicating a fourfold increase among First Nations by 2031, in contrast to a 2.3-fold increase among non-Indigenous populations (8). Notably, Cree First Nations communities in Northern Ontario reported a dementia mortality rate of 7%, in contrast to 23% for Ontario overall (9). In the U. S., a study by Apostolou et al. (10) reveals that 14% of individuals aged 45 to 64 diagnosed with Alzheimer’s disease and related dementias (ADRD) perceive this as an indicated high rate of early-onset dementia. Additionally, it is estimated that in 2020, approximately 38,000 American Indian/Alaska Native (AI/AN) individuals aged 65 and older were living with ADRD, a figure projected to double by 2030 and quadruple by 2050 within AI/AN communities (10). Another Strong Heart Study (2024) found that 54% of American Indians aged 65 and older experience cognitive impairment, and 10% have dementia across the United States (11).

These types of disparities stem from a combination of factors, including social and health legacies of colonialism, systemic racism, and insufficient access to care. Additional factors contributing to this crisis include social ecological factors such as poverty, inadequate housing, unsafe environments, isolation, lack of educational opportunities, and food insecurity. All these factors constitute what the WHO classified as social determinants of health. These factors contribute to health inequalities and differences in projections of dementia risk as well (12). However, community initiatives can reduce health disparities and lower dementia risk across the life course, promoting brain health equity (13). Addressing these issues requires an urgent political commitment to health equity funding policies.

This paper also argues that policymakers and scholars informally understand the roles and practices of Indigenous traditional healers in providing holistic dementia care and why this review considered them “unsung heroes.” In addition, and most importantly, this review notes how the WHO conceptualizes but does not operationalize the role of traditional healers in dementia care within national dementia implementation plans. Although WHO recognizes the importance of traditional healers, there are conceptual and practical barriers preventing a synergistic understanding between traditional healers and clinicians for integrated biomedical and traditional medicine in dementia care for the Indigenous population. The WHO recognized traditional healers as “community stakeholders” for dementia care and prevention globally (14, 15) to implement the WHO Global Action Plan on Dementia (2017–2025) with respectful engagement and empowerment of traditional healers as equitable partners, as there was little effort to assist integration of a traditional way of knowing system with a biomedical one. However, little existing research has been explicitly dedicated to traditional medicine and healing options that focus on dementia care and prevention for Indigenous populations. Traditional healers contribute to dementia assessment, diagnosis, and care in unique ways. They have the potential to play a catalytic role in dementia care planning and assessment with health care providers at the community level. Traditional healers offer an affordable and trusted enhancement for culturally-safe dementia care (CSDC) among diverse Indigenous communities and populations. Furthermore, there is a fundamental clash of knowledge systems that needs to be addressed.

This paper advocates that traditional healers are the “unsung heroes” who can bridge existing gaps and spearhead the creation of a compassionate, community-led dementia care model. Although the WHO (15) has formally recognized and acknowledged their role, these healers continue to be marginalized by healthcare systems that prioritize biomedical knowledge. This review, which focuses on literature from 2000 to 2025, aims to (1) analyze the systemic barriers preventing traditional healers from accessing formal care pathways, (2) highlight emerging, strengths-based models of integration that are defined and led by the community, and (3) propose a framework for equitable partnerships that respects Indigenous knowledge sovereignty and promotes CSDC.

CSDC refers to an individual treated through traditional rituals or customs instead of medical care, which means beginning the doctor-patient meeting by making the patient comfortable, listening, asking about their preferences and lifestyle, and building a human connection before starting Western medical procedures. It means respecting the patient’s concerns, preferences, and cultural values, and, if necessary, working collaboratively with healers in the patient’s culture (16). Broadly, CSDC refers to being free from all kinds of bias, racism, ageism, sexism, and other forms of discrimination; actively challenging systemic inequities; and centering the dignity and identity of those suffering from dementia and memory loss.

In Indigenous contexts, traditional healers—often referred to as Elders, knowledge holders, medicine people, and spiritual leaders—play vital roles in promoting wellness throughout the life course. This is especially true in the context of dementia care, where their contributions become increasingly crucial. Indigenous perspectives on dementia frequently diverge from biomedical understandings, viewing memory loss as a natural part of the life cycle, a spiritual transition, or a “second childhood,” rather than merely a pathological condition (8, 17–19).

Despite the legendary roles of traditional healers, the function of traditional healers as unrecognized and unsung heroes in delivering dementia care and navigation to Indigenous communities in North America has been formally acknowledged. This paper critically reviews existing literature to explore how traditional healing practices contribute to culturally safe and compassionate dementia care. Additionally, it underscores the systemic and structural barriers that continue to marginalize Indigenous knowledge within healthcare systems.

Additionally, the core purpose of this synthesis review paper is to examine the role of traditional healers in providing dementia care for Indigenous Peoples in North America, to identify how traditional healing practices contribute to CSDC, to analyze the structural and epistemic barriers that hinder the integration of traditional healing into formal healthcare systems, and to highlight promising Indigenous-led models that effectively bridge traditional and biomedical approaches through ethical collaboration and equitable reciprocal partnership. This review included various sources, including peer-reviewed journal articles, policy reports, ethnographic studies, and research led by Indigenous communities, all pertaining to traditional healing, dementia care, and Indigenous health in Canada and the United States, and published from 2000 to 2025. Priority was given to studies that emphasized Indigenous perspectives, community-based approaches, and qualitative methodologies. This review is framed through a critical lens that examines power dynamics and epistemic injustice within the healthcare system. This approach interrogates how systemic structures marginalize Indigenous knowledge systems. The literature was analyzed thematically to identify consistent findings concerning healer roles, barriers to integration, and the core components of emerging community-led care models. The synthesis prioritizes perspectives and research that are led by or conducted in deep partnership with Indigenous communities.

It also focuses on how power, political economy, and social inequality shape health experiences and the organization of healing, providing essential tools to move beyond a simplistic descriptive account of traditional healing practices (20). This approach enables a structural critique of the conditions that render traditional healers as unsung heroes. The central argument is twofold: first, the traditional healers are not just marginalized in dementia care; it is endemic, and it constitutes a form of structural violence and epistemic injustice, rooted in the colonial legacy of biomedical hegemony. Second, despite this systemic erasure, traditional healers perform indispensable, holistic work that addresses the shortcomings of biomedical dementia care, thereby playing a heroic, albeit unrecognized, role in sustaining community health and cultural integrity.

Drawing on a synthesis of scholarly literature, numerous community-based research publications, and policy documents, this paper delineates the contrasting ontological and epistemological foundations of biomedical and Indigenous understandings of dementia. Following this, the paper will present a dual analysis. The first part examines the mechanisms of systemic marginalization—the “unsung” aspect—by exploring concepts of epistemological injustice, structural violence, and biopower as they manifest in health policy, funding, and research. The second part details the vital contributions—the “heroic” aspect—of traditional healers, illustrating how they provide culturally safe, holistic care that bridges spiritual, familial, and clinical domains. Finally, the paper will discuss emergent models of collaboration and conclude with a call for a decolonized approach to dementia care that demands not only inclusion but also equitable partnership and sovereign recognition of traditional healers as essential equitable care partners and knowledge holders.

### 1.1 Historical perspectives on traditional medicine in dementia

According to the study of Liu, Wang, and Tian (21), dementia was recognized and investigated in traditional Chinese medicine (TCM). Although the word dementia is Latin in origin. The contemporary Chinese word for dementia today are referred to “Shi Zhi Zheng – (disorder with dysfunction of intelligence or loss of wisdom)”, “Ren Zhi Zhang Ai Zheng (cognitive disorder)” and “Chi Dai Zheng – (insane and idiotic)” (22), however, the famous Chinese physician Hua Tuo (140–208 AD) was the first to formally use a term for dementia and it appears in his book Hua Tuo Shen Yi Mi Zhuan during the Han Dynasty, and he prescribed a treatment for dementia. During the period of the Ming and Qing dynasties, the pathogenesis and therapy of dementia were investigated and developed as an independent subject with advanced knowledge in memory impairment, speech difficulty, sleep disorders, neuropsychiatric syndromes, and pathologic brain atrophy. These observations were renowned by Chinese physicians in ancient times (21).

Another study by Baloyannis (23) illustrated that dementia occurs in the final stages of Alzheimer’s disease and other degenerative processes of the brain cortex and subcortical centers that characterize a gradual cognitive impairment that affects mostly memory and judgment. The person with dementia is still a person who deserves to be treated with respect and dignity, respecting his/her inner life. From the viewpoint of neuro-philosophy, dementia should be considered under two different dimensions: (1) dementia as a disease, namely as a medical problem, and (2) dementia as a composite of bio-social psychological factors that affect the quality of life of the suffering human being (23).

## 2 Empirical concepts and issues

### 2.1 Ontologies: biomedical vs. Indigenous worldviews of dementia

The role of the traditional healer and the reasons for their marginalization become evident only when we recognize the significant ontological divide between Western biomedicine and Indigenous perspectives on personhood, health, and cognitive change. The biomedical model, which predominates in dementia research, diagnosis, and care in North America, is inherently materialist and reductionist. It identifies the essence of dementia—primarily Alzheimer’s disease—within the neuropathology of the individual brain. The primary neuropathological characteristics of Alzheimer’s disease are amyloid plaques and neurofibrillary tangles, with the progression of the disease indicated by a decline in cognitive and functional abilities. Medical anthropological research emphasizes that this progression is often described in deficit-based terms, leading to individuals being increasingly characterized by their losses and impairments (24). The biomedical model depicts dementia as a devastating illness, frequently described as a “living death” or a “tsunami” of pathology, narratives that typically cultivate fear, stigma, and despair, and a sense of hopelessness (25). Consequently, care within this framework focuses on pharmaceutical management, safety monitoring, behavioral control, and eventual institutionalization. In this context, the individual is primarily viewed as a patient, a site of neurological decay to be managed.

Indigenous worldviews, in contrast, are generally holistic, relational, and cyclical. Health is regarded as a state of balance and harmony, both within oneself and in relation to the entirety of creation. The Medicine Wheel, a teaching and conceptual framework used by many First Nations, symbolizes this balance among the physical, mental, emotional, and spiritual dimensions of being (26). Aging and death are acknowledged as significant components of a sacred circle. Within this ontological framework, memory loss and cognitive change are often interpreted differently. Moreover, research with diverse Indigenous communities across North America highlights several interconnected explanatory models that differ from the biomedical disease narrative (27, 28).

### 2.2 Dementia as natural aging and “second childhood”

Some communities perceive memory loss as a normal part of the aging process, a return to the beginning of the circle of life (17, 18). An Elder may be seen as entering a second childhood, requiring the same nurturing, patience, and unconditional love as an infant. Research studies are also finding that Indigenous peoples view the lifespan differently and do not demarcate chronological age periods in a linear fashion as Western human development theories do. Having a circular view of the lifespan, which balances the physical, mental, emotional, and spiritual dimensions of their being, also affects their dementia caregiving practices (17, 18). This perspective, rather than being dismissive, encourages a culturally ingrained attitude of profound respect, kindness, and attentive care.

### 2.3 Dementia as a spiritual transition

Cognitive changes may be understood as a preparatory stage for the journey to the spirit world. As the Elder “forgets” the concerns of the physical world, they are thought to be drawing closer to the ancestors and the Creator. This is viewed not as a loss but as a spiritual transformation—a shedding of ego and worldly attachment (29, 30). Care in this context is an honor, a sacred duty to support a loved one with dementia through this important transition.

### 2.4 Dementia as a social/historical etiology

In certain communities, cognitive decline is linked to the profound social and historical upheavals brought by colonialism. The intergenerational trauma stemming from residential schools, forced relocation from home territories, loss of language, and forbidding of cultural practices is viewed as spiritual wounds that may lead to cognitive and emotional decline and distress in later life (31). From this perspective, dementia is seen not merely as an individual brain disease but as a manifestation of collective historical suffering.

The Indigenous worldviews are not mutually exclusive and disrespectful in the Western dementia care approach; rather, they may coexist within communities and families. Moreover, this paper raises critical questions about what unites them both, the clinician and the healer: a fundamental shift in focus from pathology and deficit to relationship, meaning, and continuity. The primary question transforms from “How do we manage this disease?” to “How do we honor and support this person on their journey?” This ontological chasm highlights the cultural incongruity that Indigenous families encounter when navigating dementia. It also delineates the space in which the traditional healer operates—a realm that the biomedical system, by its very design, fails to understand, including its social norms and ethical values.

The role and experiences of traditional healers in dementia care are shaped by their Indigenous knowledge, land, language, culture, ancestral and sacred teachings, and their connection to the cosmic spirit. Charlotte Reading (32) magnificently illustrates the structural determinants of the Aboriginal people’s health as an Indigenous determinants of health framework using the metaphor of a tree. This framework acknowledges the historical legacies within settler-dominated societies that have distal, deeply rooted influences, resulting in complex and interconnected impacts on the intermediate (trunk) and proximal (crown and leaves) determinants of Indigenous peoples’ health, and suggests that the deeply embedded roots of Indigenous worldviews, spirituality, and self-determination are mirrored in both the historical and contemporary structures of the distal determinants (32). Furthermore, the framework emphasizes the significance of relationships, kinship, language, and cultural traditions to inform diverse dementia care practices that focus on spiritual and emotional wellness provided by traditional healers.

### 2.5 Power dynamics and relationship between biomedicine and traditional healing in dementia care

Traditional healers and healthcare providers navigate intricate power dynamics, necessitating the formation of equitable partnerships, the cultivation of humility in collaboration, and the assurance of knowledge ownership. Despite these dynamics, there exists a shared vision for dementia care. This approach facilitates the integration of traditional healing practices into dementia care, recognizing and honoring the insights of traditional healing practitioners and Elder knowledge-holders. Additionally, it aims to enhance efforts to decolonize dementia care by increasing access to traditional healing methods. Furthermore, these power dynamics can create pathways for emerging Indigenous researchers and traditional healing practitioners to come together, promoting the appreciation of the diversity of traditional healing as land-based medical knowledge and practices. This integration aims to merge biomedical and traditional medicine in the context of dementia care, relying on the relational dimensions of healing that encompass the mind, body, and spirit. Furthermore, the knowledge that comes from ancestral oral history and non-scriptural knowledge and guidance from Elders, traditional healers, knowledge-holders, community members, and leaders is greatly acknowledged and recognized with humble gratitude and honor.

## 3 Policy implications and research gaps

### 3.1 The urgent context: rising prevalence and cultural difference

Indigenous populations in North America are experiencing a disproportionate increase in dementia prevalence, a prioritizing public health issue driven by social determinants of health and historical inequities (7, 8). Beyond the epidemiological data, the central challenge lies in a clash of understanding. Numerous Indigenous communities perceive dementia as an integral aspect of their life cycle concepts. The connection between traditional healing and Indigenous knowledge in dementia care is important. Jacklin and Pitawanakwat (33) discussed the concept of the circle of life, which is integral to Indigenous knowledge about dementia within Anishinaabe First Nations in Canada. This highlights that traditional healing practices and cultural medicine cannot be separated when addressing dementia care in Canadian Indigenous communities. Pitawanakwat, a community health researcher, conveyed her message powerfully:

“I want to introduce the concept of the circle of life as it relates to four of our seven grandfather teachings. Our Elders are living in their seventh stage of life. They have a lifetime of wisdom that is respected. Interactions with an elder instinctively show kindness and love. We are a humble people who value our time walking here upon the earth as a spiritual collective to learn and share for the benefit of seven generations who will walk the same earth ahead of us. In an Elder’s last stage, they are transitioning back into the spiritual world, part of the continuum of life” (33).

This perspective recognizes the individual’s dignity and spiritual importance and fundamentally opposes the biomedical model that emphasizes deficiency and decline (28). Such cultural incongruity poses a significant barrier to accessing and trusting mainstream dementia services.

### 3.2 Traditional healers as custodians of compassionate, holistic care

Traditional healers offer a holistic, strengths-based approach that effectively addresses gaps in the biomedical model. Their roles, which are essential for delivering compassionate care, encompass.

#### 3.2.1 Spiritual and ceremonial care

Leading ceremonies such as smudging, sweat lodges, and prayer to provide comfort, facilitate spiritual transitions, and connect individuals with their ancestors, community, and creator (30).

#### 3.2.2 Cultural nourishment and identity affirmation

Employing language, storytelling, song, symbols, and drumming to uphold personhood and cultural identity, which are vital for well-being amidst cognitive changes.

#### 3.2.3 Family and community guidance

Providing counseling and support to families navigating the dementia journey, frequently utilizing foundational teachings like the Medicine Wheel or the Seven Grandfather Spiritual Teachings to honor care as an expression of love, responsibility, and reciprocity.

#### 3.2.4 Application of medicinal knowledge

Utilizing plant-based medicines to promote physical comfort, cognitive support, and holistic balance. This approach shifts the perspective of care from “managing a decline” to “honoring a journey.” It aligns with a compassionate philosophy that views the person as more than a diagnosis, thereby offering a model of dignity that is urgently needed (29).

#### 3.2.5 Systemic barriers: the structural marginalization of traditional healers

Despite their crucial role, traditional healers are systematically excluded from integrated dementia care pathways. This marginalization is both structural and multifaceted.

#### 3.2.6 Epistemological injustice and policy exclusion

Healthcare systems rely on licensing, funding, and accreditation models that recognize only biomedical credentials. Indigenous knowledge, which is transmitted orally and spiritually, is frequently classified as “unscientific” or “alternative.” As a result, healers are often excluded from formal referral systems, sustainable funding opportunities, and inclusion on clinical care teams (34).

In terms of epistemological injustice in law, policy, and practice, the critical mechanism of epistemic injustice denies and marginalizes individuals (35). Testimonial injustice occurs routinely when health administrators disregard healers’ assessments or when clinicians dismiss family requests for ceremonial spaces as non-essential. In these situations, the healer’s testimony is not considered credible evidence in care planning and is not considered for health policy frameworks, hospital intake forms, and diagnostic criteria due to a lack of conceptual categories for terms such as “spiritual transition,” “spiritual pathways,” or “balance restoration.” This absence of shared hermeneutical resources forces Indigenous families to either abandon their frameworks or risk being misinterpreted as experiencing denial or superstition within clinical environments and spheres.

#### 3.2.7 Power imbalances and clinical skepticism

Ongoing power dynamics in healthcare can foster clinicians’ skepticism toward traditional practices. Meanwhile, communities may develop distrust toward clinical settings that are perceived as culturally unsafe or disrespectful of their worldviews (27, 28). This mutual distrust disrupts the provision of care.

#### 3.2.8 Structural violence through political economy

The landscape of healthcare funding and delivery enacts structural violence against traditional healing systems. In Canada, for instance, provincial health insurance plans lack billing codes for traditional healers. Hospitals and long-term care homes, which are primarily funded for biomedical services, rarely budget for hiring healers or establishing permanent ceremonial spaces, such as medicine lodges or safe spaces for Indigenous older adults living with dementia, even in hospitals (36). Additionally, research funding from major organizations overwhelmingly prioritizes medical and pharmaceutical research over community-based, participatory research led by Indigenous scholars and healers. This economic marginalization represents a form of violence inherent in neoliberalism, systematically denying resources and relegating traditional care to an informal, volunteer-dependent supplement, rather than recognizing it as a core, funded component of health services.

In terms of infrastructure for collaboration, there is a significant lack of formal protocols, dedicated funding, and designated roles to support collaboration between health authorities and traditional healer councils. Furthermore, the denial of funding constitutes a breach of treaty obligations.

#### 3.2.9 The mechanisms of marginalization

The crucial contribution of healers is often invisible on the radar of the mainstream healthcare system. The “unsung” status of traditional healers is not merely a passive oversight; it results from a healthcare system shaped by colonial history and maintained by contemporary power structures as well as operating costs and national/local policies embedded within the history and structures. For instance, clinicians, including physicians and geriatricians, often reject the validity of traditional healing and alternative medicines. This rejection complicates collaborative care for Indigenous patients with dementia (37). Both traditional and Western medicine, along with non-Western practices, contribute uniquely to the development of spiritual and culturally based dementia care plans and guidelines. These approaches may encompass mindful meditation, art, music, dance, sacred fire, sweat lodges, medicine wheels, and ritual ceremonies.

### 3.3 Emerging community-led models of integration

Recent literature indicates a promising shift toward community-defined and community-led models that actively involve traditional healers as partners (34). These models represent grassroots collaborations rather than top-down integrations. Furthermore, the role of traditional healers in supporting the resilience and care of family and community caregivers is not well covered in the academic literature but is a critical component of integrating the gifts of traditional healers in dementia care (34).

#### 3.3.1 Providing authentic culturally-safe dementia care

Cultural safety transcends cultural sensitivity, which merely acknowledges differences. It aims for an outcome in which the recipient of care feels their identity and worldview are genuinely respected and protected. Traditional healers inherently practice cultural safety. They implement CSDC by conducting assessments and interventions in the patient’s preferred language, involving family and community members in decision-making, and employing spiritually meaningful practices. These healers foster a safe environment where dementia is not regarded as a shameful secret but rather as a shared experience that can be addressed through collective resources and cultural protocols. This approach sharply contrasts with clinical settings that may feel alienating, instill fear, and dismiss Indigenous identity.

In terms of CSDC models, the pioneering efforts led by Indigenous Elders in Northern Ontario have developed and validated community-based CSDC models (34). These models offer a practical framework for establishing joint advisory councils that unite traditional healers, Elders, family caregivers, and biomedical providers to collaboratively design care plans. They embody the “Two-Eyed Seeing” principle, which integrates the strengths of Indigenous and Western knowledge systems side by side.

In Hawaii, the Papa Ola Lokahi (POL) native community is dedicated to enhancing the brain health of Kupuna—older adults aged 65 and older experiencing Alzheimer’s Disease and Related Dementias (ADRD). The POL is implementing an action plan, a Native Hawaiian Healthy Brain Roadmap to better understand ADRD’s impact on Native Hawaiians (38). This roadmap prioritizes integrating Kupuna healing practices and the knowledge of cultural masters (including traditional healers), which are essential to promoting awareness and support for Native Hawaiian healing traditions. These practices are deeply intertwined with the community’s cultural, spiritual, and historical identity, underscoring the importance of respecting and honoring Kupuna wisdom within their cultural, linguistic, and land-based medicinal context.

#### 3.3.2 Land-based and ceremony-led programs

There is a growing emphasis on interventions that are firmly rooted in cultural practices. Research is confirming the effectiveness of programs that incorporate land-based healing, traditional foods, and ceremonial elements as central therapeutic components, which aim to prevent cognitive decline and support individuals living with dementia (39).

- *Principles of successful community-led models*: An analysis of these initiatives reveals several common, non-negotiable principles.
- *Community ownership and co-design*: Initiatives are led by the community from inception to evaluation.
- *Respect for knowledge sovereignty*: Indigenous knowledge is acknowledged as a distinct, valid, and autonomous system.
- *Relational accountability*: Partnerships maintain accountability to the community and its governance structures.
- *Sustainable, ethical resourcing*: Healers receive equitable compensation for their expertise and time as reciprocity and honorarium.

### 3.4 Healing historical and intergenerational trauma

The biomedical model is often ill-equipped to address the impact of historical trauma on health. For many Indigenous Elders and healers, the symptoms of dementia can evoke traumatic memories related to residential schools or other forms of colonial violence. A healer, who operates within a framework that acknowledges this historical context, can effectively address these wounds through ceremonies, counseling, and the reaffirmation of cultural identity. This trauma-informed healing and care, which is rooted in Indigenous epistemology, represents a vital aspect of care that biomedicine cannot replicate and repair.

### 3.5 Biopower and evidence-based medicine approach

The concept of biopower elucidates how this marginalization is legitimized. The state, through medical licensing boards and institutions, authorizes specific forms of knowledge as scientifically valid (40). The gold standard of “evidence-based medicine” (EBM)—typically represented by randomized controlled trials—serves as a gatekeeping mechanism. The complex, holistic, and spiritual outcomes of traditional healing practices (e.g., an increased sense of peace, strengthened family connections, and cultural continuity) are challenging to quantify and conform to the metrics demanded by EBM. Consequently, these healing practices are often labeled as “anecdotal,” “traditional,” or “cultural,” terms that, within the biopolitical hierarchy, position them beneath “scientific” biomedicine. This labeling exerts biopower by defining what is considered legitimate treatment, thereby indirectly coercing communities to conform to the biomedical model to access essential services.

### 3.6 Elimination in research and discourse

Despite the evident disparities in dementia care, most of the research on Indigenous dementia in Canada and the U. S. has predominantly concentrated on epidemiology, caregiving burdens, and the adaptation of Western tools (11, 27, 41). While this paper is significant, it frequently underrepresents the specific roles, knowledge, and effectiveness of traditional healers. Instead, they are often relegated to footnotes or described merely as a “cultural consideration,” rather than being recognized as primary agents or care partners in care and healing. This scholarly erasure perpetuates their undervalued status and fails to establish the academic foundation necessary to challenge existing policy barriers. Consequently, the cumulative impact of these mechanisms creates a healthcare environment in which traditional healers are, at best, tolerated as a cultural accommodation and, at worst, actively excluded. They are forced to operate heroically outside the system precisely because the system is inherently designed to neglect and underestimate them.

### 3.7 The indispensable contribution

In the context of systemic marginalization, traditional healers continue their genuine support by providing ethical and dignified care that is not only complementary but often essential for wellness. Their heroism is evident in their ability to address the significant deficiencies of the biomedical model for Indigenous patients with dementia. This remains true despite physicians rejecting the validity of traditional healing and alternative medicines, as well as the challenges faced in the failure of collaborative care for Indigenous dementia patients in the First Nations communities of southwestern Ontario (37).

### 3.8 Bridging explanatory models and reducing stigma

When families perceive an Elder’s confusion or forgetfulness as part of a spiritual journey, it can shift the approach to care from a burden to a sacred responsibility. Healers validate these alternative explanations within the community, helping to alleviate internalized stigma and fear. They also serve as a vital bridge between families and clinical providers, translating family concerns into a language the medical team can understand, and vice versa. This fosters a more collaborative and less adversarial relationship with the healthcare system.

### 3.9 Sustaining caregivers and community resilience

The burden on family caregivers in Indigenous communities is significant, exacerbated by geographic isolation and limited resources. Traditional healers provide support not only to individuals with dementia but also to the entire caregiving network and local community support systems. Ceremonies offer caregivers spiritual strength and moments of respite. The presence of the healer affirms their efforts and reinforces the community’s values of collective care, helping to prevent burnout and sustain the informal care system that many Indigenous people depend on. Traditional healers also engage with the local biologists of dementia (24), which refers to the culturally shaped experiences and expressions of cognitive change stemming from a specific history of colonization, resilience, and spiritual beliefs. This holistic and contextually grounded perspective is commendable, as it nurtures life, meaning, and connection in the face of a condition that biomedicine often defines solely in terms of loss.

## 4 Discussion: envisioning a compassionate, community-led dementia-friendly culture

The analysis confirms that traditional healers are not merely supplemental providers but essential leaders in reimagining dementia care for Indigenous peoples. A truly compassionate, community-led model must be built on foundations that address historical harm and promote equitable power sharing. Furthermore, recognizing the invaluable contributions of traditional healers is the initial step in fostering equitable partnerships to create Indigenous traditional healing and medicine within community models of dementia care, addressing significant challenges in a decolonizing future. It is essential to transform systems so that their work is celebrated, supported, and integrated as a matter of equity rather than as an exception. While emerging models indicate a promising future, considerable challenges persist.

### 4.1 Foundational pillars for equitable partnership and cultural humility

It is crucial to advance with a paradigm shift that is underpinned by key pillars, including

- *Cultural safety and humility as mandates:* Healthcare systems and professionals must engage in mandatory, ongoing training focused on cultural safety and humility. This involves critical self-reflection on issues of power and bias, alongside a commitment to fostering environments where Indigenous patients and healers feel respected and safe.
- *Formalizing power-sharing structures*: Sustainable integration requires the establishment of formal, resourced mechanisms for partnership. This includes memoranda of understanding between health authorities and traditional healer councils, as well as the creation of healer positions within community health teams.
- *Implementing enabling policies*: Systemic change demands concrete policy actions, such as developing billing codes for healer services, establishing cross-cultural care protocols, and providing funding for community-led research and program implementation.

### 4.2 The community-led dementia care model in practice

In this proposed model, the traditional healer is an integral member of the care ecosystem. Another notable initiative is the Canadian Indigenous Cognitive Assessment (CICA), a dementia screening tool co-created with Indigenous communities to ensure cultural appropriateness. This tool underwent adaptation, piloting, and reliability and validity testing within Anishinaabe communities on Manitoulin Island. While adapting, refining, and testing the CICA tool, traditional healers were not included in its practical implementation (34). This lack of integration may lead to a disconnect between the assessment and the community’s cultural practices, ultimately affecting the effectiveness of dementia care, as traditional healers possess valuable insights and methods crucial to culturally grounded care. However, the path to equitable partnership identifies several barriers and pathways:

- *Care planning*: This process is collaborative, combining the clinical care plan with a healing plan that may encompass ceremonies, traditional diets, language revitalization activities, and family support through sharing circles.
- *Caregiver support*: This support is provided within a cultural framework that respects the caregiver’s role, mitigates feelings of isolation through community engagement, and offers respite grounded in cultural understanding.
- *Health promotion and prevention*: This aspect is guided by community knowledge of protective factors, which include connections to the land, language, ceremonies, and traditional foods.

### 4.3 Two-eyed seeing culturally-safe dementia care policy and model

One promising research framework is “Two-Eyed Seeing” (Etuaptmumk), a concept from the Mi’kmaw culture as articulated by Elder Albert Marshall. This framework encourages individuals to view the world from two perspectives: one eye represents the strengths of indigenous knowledge and ways of knowing, while the other eye represents the strengths of Western knowledge and ways of knowing. By using both perspectives in tandem, the goal is to benefit all communities involved (42). This approach promotes a synergistic partnership that honors the integrity of both systems rather than advocating for assimilation.

The practical applications of this concept are beginning to emerge. Shrestha et al. (34) created, tested, and validated CSDC models in collaboration with Elders, traditional healers, and knowledge holders from the Northern Ontario (34). These experts were part of a local community advisory group for dementia research on Manitoulin Island, which also included two Elders affiliated with Laurentian University’s School of Indigenous Relations in the Greater Sudbury Area and Georgian Bay. The CSDC models serve as blueprints for establishing community advisory councils in which traditional healers, Elders, clinicians, and family caregivers collaborate as equals to design and develop a roadmap for local dementia care.

Furthermore, this model exemplifies a two-eyed seeing approach that merges Indigenous and Western medical perspectives. It encourages collaboration between clinicians and local Indigenous healthcare partners, integrating traditional healers, Elders, and knowledge holders into the development of culturally tailored education and training for biomedical healthcare providers. Additionally, it advocates for holistic interventions, including land-based healing, ceremony-based care, and trauma-informed support. Although the model shows promise, its implementation encounters considerable challenges; most notably, it necessitates structured facilitation by trained staff. Moreover, it aspires to create a supportive, nurturing environment that fosters healing relationships among Elders, knowledge holders, traditional healers, clinicians, team members, and policymakers.

#### 4.3.1 The risk of tokenism

Inviting a healer respectfully to participate in a committee without granting genuine decision-making power or allocating resources could be a superficial gesture. True integration requires shared vision, mission, authority, ownership, and equitable partnership rather than just consultation.

#### 4.3.2 Protecting knowledge sovereignty

Indigenous healing knowledge is sacred and is often governed by specific protocols. It is essential that the process of integration does not result in the exploitation, commodification, or appropriation of this knowledge by non-Indigenous practitioners or researchers. Ethical guidelines should be defined by the community.

#### 4.3.3 Overcoming logistical barriers

Addressing practical challenges and barriers, such as sustainable funding for healer honoraria, which should be viewed as reciprocity and a gift, is essential. Additionally, establishing dedicated ceremonial spaces within health facilities and resolving liability and insurance issues genuinely require political will, political commitment, and innovative policy solutions.

Furthermore, this entails actively dismantling the colonial power structures within healthcare that uphold biomedical hegemony. It also involves supporting Indigenous communities in the process of rebuilding their health governance models, wherein traditional healers work in partnership with clinicians. For instance, it necessitates a fundamental rethinking of what constitutes “evidence,” expanding this concept to encompass narrative, relational, and spiritual outcomes that are valued by the communities themselves. Additionally, it requires the allocation of resources directly to local Indigenous organizations, enabling them to develop and deliver dementia care according to their terms and conditions with full autonomy and respect. The healer’s insights into the individual’s spiritual, familial, and community life enhance the assessment process, which utilizes tools such as the Canadian Indigenous Cognitive Assessment.

There is a critical challenge to build mutual trust and reciprocity to educate, engage, and empower traditional healers, caregivers, Indigenous people living with dementia, Indigenous community care partners, and community leaders. Within this education, engagement, and empowerment it is important to bridge research, education, and practice innovation with dementia equity, addressing bias and equity gaps through co-design and co-learning among themselves about dementia education and brain health. For instance, traditional healers can offer valuable insights and educational resources about dementia and brain health while respecting cultural values and maintaining ethical norms and spiritual pathways.

Through this process, Indigenous community members, Elders, traditional healers, knowledge holders, and healthcare providers collaboratively identify priorities, negotiate culturally relevant care components, and define what constitutes an Indigenous-friendly dementia care plan model and navigation program. Key elements often include integrating traditional foods, Indigenous languages, and cultural and spiritual practices and pathways, as well as complementary biomedical and traditional healing methods. Consultation with Elders and traditional healers is foundational to aligning service delivery with Indigenous worldviews and community-specific values. This approach emphasizes continuous engagement and relational accountability, ensuring that community partners remain involved throughout the planning, implementation, and evaluation processes. Importantly, it allows Indigenous communities to articulate their success indicators and principles for CSDC.

### 4.4 Limitations

The emerging body of literature documenting the roles of healers in dementia care is still in its infancy, reflecting their historical exclusion from this field. This review focuses primarily on Indigenous communities in Canada and the United States and does not include extensive primary data collection. While it draws on qualitative literature, further longitudinal and participatory research led by Indigenous communities is needed to strengthen the evidence base. Consequently, this limits the scope of this review. Further suggestions should include:

- *Community-based participatory research (CBPR)*: CBPR is a research method to collaborate with community members, stakeholders, and researchers. This approach should prioritize research led by Indigenous communities, concentrating on the effects of healer-integrated dementia care models on wellness, caregiver burden, and healthcare utilization through ethnographic observation.
- *Longitudinal studies:* These studies should track the long-term effects of community-led dementia care on cognitive, spiritual, and cultural health indicators.
- *Resource development:* This is essential to support the creation of training resources and curricula in local Indigenous languages, establish ethical guidelines for collaboration, and develop advocacy tools for communities aiming to create their own care models. Oversampling high-American Indian/Alaska Native-population states in public health research builds American Indian/Alaska Native research capacity.

## 5 Conclusion

The traditional healer, as the “unsung hero” of Indigenous traditional healing and medicine in dementia care, represents a figure shaped by a specific historical and political context—the ongoing colonial encounter in North America. This paper illuminates that their status arises from active processes of epistemic and legal injustice, structural violence, and biopower exercised by a healthcare system that disregards Indigenous ways of knowing. Nonetheless, within the void created by this systemic failure, healers engage in heroic and essential, genuine work. They offer culturally safe, holistic, and spiritually grounded care that addresses the limitations of the biomedical model, heals historical trauma, and supports families and communities. Without the engagement and empowerment of traditional healers in the screening and diagnosis process of dementia, the implementation of the WHO Global Action Plan (2017–2025) on the public health response to dementia will be hindered, as these traditional healers are essential community stakeholders and allied partners for dementia care and prevention worldwide. Celebrating their heroism without confronting the underlying structures that necessitate it is inadequate. The call to action is explicit: we must shift from rendering healers to recognizing them as sovereign knowledge holders and equitable partners. Such recognition demands a decolonizing project—a fundamental transformation in health policy, research, education, and funding to respect Indigenous self-determination in health. It represents achieving true reconciliation and health equity for Indigenous people living with dementia. This paper suggests further research on ethnographic observational community-based participatory action research on the CSDC models in Indigenous communities in both Canada and the U. S. Additionally, empowering and fundamental medical education for Indigenous traditional healers can develop a bio-cultural model of dementia care to enhance holistic wellness for people with dementia and memory loss through a community-based support system, spiritual pathways, dementia-friendly environment, dietary and language preferences, and Indigenous arts, crafts, music, sciences, and philosophy.

In terms of future studies, the CDC and Alzheimer’s Association have released two public health roadmaps for public health action in Indian Country, which have launched at least seven US federal government funding streams for tribal action on dementia since 2019 (43) Further study of the role of traditional healers and practices in these initiatives would be worthwhile.
